# Inflammatory stress exacerbates ectopic lipid deposition in C57BL/6J mice

**DOI:** 10.1186/1476-511X-10-110

**Published:** 2011-06-30

**Authors:** Mei Mei, Lei Zhao, Qing Li, Yaxi Chen, Ailong Huang, Zac Varghese, John F Moorhead, Suhua Zhang, Stephen H Powis, Qifu Li, Xiong Z Ruan

**Affiliations:** 1 Centre for Lipid Research, Key Laboratory of Molecular Biology on Infectious Diseases, Ministry of Education, The Second Affiliated Hospital, Chongqing Medical University, P.R.China; 2 Department of Endocrinology, First Affiliated Hospital, Chongqing Medical University, P.R.China; 3 John Moorhead Research Laboratories, Centre for Nephrology, University College London (UCL) Medical School, Royal Free Campus, University College London, UK

**Keywords:** sterol regulatory element binding protein1, fatty acid synthase, acetyl CoA carboxylase, adipose triglyceride lipase

## Abstract

**Background:**

Chronic systemic inflammation and abnormal free fatty acid metabolism are closely related to ectopic lipid deposition. In this study, we investigate if inflammation tissue-specifically disrupts lipogenesis and lipolysis in nonadipose tissues and adipose tissue, resulting in ectopic lipid deposition in C57BL/6J mice.

**Methods:**

We used casein injection in C57BL/6J mice to induce a chronic systemic inflammatory stress in vivo. Serum was analyzed for free fatty acid and cytokines. Insulin sensitivities were evaluated by glucose and insulin tolerance tests. Liver, muscle, adipose tissues were taken for lipid analysis. Real-time polymerase chain reaction and western blotting were used to examine the gene and protein expression of molecules involved in adipogenesis and lipolysis in tissues.

**Results:**

Casein injection elevated serum levels of IL-6 and SAA in mice, which are associated with increased lipid accumulation in liver and muscle, suggesting that chronic systemic inflammation induces ectopic lipid deposition in nonadipose tissues. The inflammatory stress upregulated mRNA and protein expression of sterol regulatory element binding protein 1, fatty acid synthase, and acetyl CoA carboxylase alpha, while inhibited these molecules expression in adipose. Interestingly, in the same experimental setting, inflammation increased triglyceride lipase and hormone-sensitive lipase expression in white adipose tissue. Inflammation also induced insulin resistance and increased serum free fatty acid levels in C57BL/6J mice.

**Conclusions:**

Chronic systemic inflammation increased lipogenesis in nonadipose tissues and lipolysis in white adipose tissue, resulting in ectopic lipid deposition in nonadipose tissues. This disturbed free fatty acid homeostasis and caused insulin resistance in C57BL/6J mice.

## Background

Ectopic lipid deposition which is defined as excess lipids deposited in nonadipose tissues, mainly in liver and skeletal muscle, has been indicated to be a possible major culprit in development of insulin resistance and is rapidly becoming a world-wide public health problem because of the associated development of type 2 diabetes (T2DM), non-alcoholic fatty liver disease (NAFLD) and cardiovascular disease [1]. Using magnetic resonance spectroscopy, it has been demonstrated that increased intrahepatic lipid content is closely correlated to impaired hepatic insulin sensitivities in nondiabetic and diabetic individuals [2,3]. Consistently, in T2DM patients, insulin requirements are closely related to their hepatic lipid content [4]. Studies also found that an increase of intramyocellular lipid content, assessed by muscle biopsy or magnetic resonance/computed tomography, was positively correlated with the development of muscle insulin resistance and a better predictor of impaired insulin action than visceral adiposity [5], while a decrease in intramyocellular lipid content by weight loss improved insulin sensitivity [6,7].

It has been demonstrated that in both patients and animal models, chronic systemic inflammation characterized by the increase of serum levels of C-reactive protein (CRP) and pro-inflammatory cytokines, such as tumor necrosis factor alpha (TNF-α), and interleukin-6 (IL-6) is closely related the development of NAFLD, insulin resistance and other metabolic disorders [8]. Furthermore, *in vivo *and *in vitro *studies have demonstrated that inflammatory cytokines increase serum free fatty acid (FFA) levels, causing dyslipidemia and NAFLD which can be prevented by using anti-cytokine antibodies in a mouse model [9,10]. However, the effect of chronic systemic inflammation on FFA accumulation in liver and muscle as well as its underlying mechanisms remains unclear.

Increasing lipogenesis in nonadipose tissues play a crucial role in ectopic lipid deposition [11]. In mammalian species, lipogenesis of cell is under control of a family of membrane-bound transcription factors designated sterol regulatory binding proteins (SREBPs). SREBPs are members of the basic helix-loophelix leucine zipper family of transcription factors [12,13], which contain two transmembrane domains and localize in the endoplasmic reticulum (ER) as a precursor protein after synthesis. SREBPs on demand are escorted to the Golgi complex for two sequential cleavages by two proteases, by which the transcriptional active domains (NH2- terminal) enter the nucleus and bind to the cognate sterol-regulatory element (SRE) sites, activating transcription of target genes. Two forms of SREBPs have been characterized: SREBP1 and SREBP2. Specific analyses suggest SREBP1 may be selectively involved in activation of genes in free fatty acid metabolism. SREBP1 regulates the expression of genes encoding rate-limiting enzymes responsible for *de novo *lipogenesis, of which fatty acid synthase (FAS) and acetyl-CoA carboxylase alpha (ACCα) seems to be particularly important since their expressions are parallel to the changes of FFA synthesis rates.

FFA homeostasis of adipocytes is not only dependent on lipogenesis but also lipolysis. Both adipose triglyceride lipase (ATGL) and hormone-sensitive lipase (HSL) can initiate triglyceride (TG) degradation by cleaving the first ester bond. Importantly, ATGL represents the rate-limiting lipolytic enzyme in mammals, and HSL, increased by insulin resistance is unique in its capacity to break down the second ester bond, converting diglycerides (DG) to monoglycerides in adipose tissue.

We and other groups demonstrated that chronic systemic inflammation causes cholesterol accumulation in liver, kidney and vessels by causing cholesterol redistribution and disrupting intracellular cholesterol homeostasis [14]. The current study was undertaken to investigate if chronic systemic inflammation activated SREBP1, FAS and ACCα expression in liver and muscle, and increased ATGL and HSL expression in white adipose tissue, thereby causing ectopic lipid deposition as well as dyslipidemia and insulin resistance in C57BL/6J mice.

## Methods

### Animal Model

Animal care and experimental procedures were performed with approval from the Animal Care Committee of Chongqing Medical University. Eight week-old C57BL/6J mice were randomly assigned to receive a normal chow diet (NCD, n = 6) or NCD plus subcutaneous injection of 0.5 mL 10% casein (NCD+Casein, n = 6) or a high fat diet (15% fat, 1.25% cholesterol, 0.5% cholic acid) (HFD, n = 6) or HFD plus subcutaneous injection of 0.5 mL 10% casein (HFD+Casein, n = 6). Subcutaneous injection of 0.5 mL PBS was used as controls in the NCD and HFD groups. The injections were done every other day and the mice were culled 18 weeks after first injection. At termination, blood samples were taken for cytokines and lipid assays, and tissue samples were collected for further assessments.

### Serum Analysis

Serum IL-6 (R&D, Minneapolis, USA) and serum amyloid A protein (SAA) concentrations were measured by ELISA kits. Intracellular TG concentrations were determined enzymatically with commercial kits (Jiancheng, Nanjing, China). FFA concentrations were determined calorimetrically using commercial kits (Applygen Technologies, Beijing, China).

### Observation of Lipid Accumulation

The lipid accumulation in liver, muscle and white adipose tissue (epididymal fat pads) of C57BL/6J mice was evaluated by Oil Red O staining. Briefly, samples were fixed with 5% formalin solution and then stained with Oil Red O for 30 minutes. Finally, the samples were counterstained with hematoxylin for 5 minutes. Liver, muscle and white adipose tissue were fixed, dehydrated, infiltrated and embedded following routine method. Results were examined by light microscopy.

### Quantitative Measurement of Intracellular TG and FFA

Quantitative measurements of TG and FFA were performed using commercial kits (Jiancheng, Nanjing, China and Applygen Technologies, Beijing, China). Briefly, samples were collected and lipids were extracted by addition of 1 mL solvents (TG: isopropanol/chloroform =2/3.5, FFA: chloroform/heptane/methanol = 5/5/1). The lipid phase was collected, dried in vacuum. The concentration of TG and FFA were analyzed using standards and normalized by total protein from tissues.

### Glucose Tolerance Tests

Before each glucose tolerance tests, mice were starved overnight but allowed free access to water. The glucose tolerance was tested by the intraperitoneal injection of 2 mg D-glucose/g body weight (Sigma, St. Louis, US). The blood glucose and serum insulin concentrations were determined in blood, which were taken from the cut tail tip, before and 15, 30, 60, and 120 minutes after the administration of glucose. The glucose concentrations were determined using an ACCU-CHEK Advantage blood glucose meter (Roche, Mannheim, Germany).

### Insulin Tolerance Tests

Before each insulin tolerance tests, mice were starved overnight but allowed free access to water. The insulin tolerance was tested by the intraperitoneal injection of 1 mU insulin/g body weight (Sigma, St. Louis, US). The glucose concentrations in blood were determined using an ACCU-CHEK Advantage blood glucose meter (Roche, Mannheim, Germany), which were taken from the cut tail tip, before and 15, 30, 60, and 120 minutes after the administration of insulin.

### Real-time Reverse Transcription Polymerase Chain Reaction

Total RNA were isolated from liver, muscle and white adipose tissue homogenates from C57BL/6J mice using the guanidinium-phenol-chloroform method. Real-time reverse transcription polymerase chain reaction (PCR) was performed in a Bio-Rad Sequence Detection System (Hercules, US) using SYBR Green dye (Applied Biosystems Inc, Foster City, US) according to the manufacturer's protocol. All the primers (Sigma-Genosys, UK) were designed by Primer Express Software V2.0 (Applied Biosystems, UK) (Table 1). To normalize expression data, 18s rRNA was used as an internal control gene.

**Table 1:** Mouse TaqMan Primers for Real-time PCR

Genes	Mouse TaqMan Primers
SREBP1	5'-GCCCACAATGCCATTGAGA-3' sense
	5'-CAGGTCTTTGAGCTCCACAATCT-3' antisense
FAS	5'-CCTGGATAGCATTCCGAACCT-3'sense
	5'-AGCACATCTCGAAGGCTACACA-3'antisense
ACCα	5'-CGCTCAGGTCACCAAAAAGAAT-3'sense
	5'-GTCCCGGCCACATAACTGAT-3' antisense
ATGL	5'-CCTCAGGACAGCTCCACCAA-3'sense
	5'-TTGAACTGGATGCTGGTGTTG-3'antisense
HSL	5'-CGAGACAGGCCTCAGTGTGA-3'sense
	5'-GAATCGGCCACCGGTAAAG-3'antisense
18s rRNA	5'-TCGAGGCCCTGTAATTGGAA-3' sense
	5'-CCCTCCAATGGATCCTCGTT-3' antisense

### Western blotting

Cytoplasm and nuclear proteins were extracted from liver, muscle and white adipose tissue using commercial kits (Pierce, Rockford, US). Sample proteins were separated by Sodium dodecyl sulfate polyacrylamide gel electrophoresis in a Bio-Rad mini Protein apparatus, then were transferred to NC membranes (Amersham Bioscience, Piscatway, US). The membrane were blocked with blocking buffer, and incubated with primary antibodies (anti-SREBP1, anti-FAS, anti-ACCα , anti-ATGL, anti-HSL and anti-β-actin from Santa Cruz biotechnology, Inc., Santa Cruz, CA), followed by an incubation with horseradish peroxidase-labeled secondary antibodies. Finally, detection procedures were performed using ECL Advance Western Blotting Detection kit (Amersham Bioscience, Piscatway, US).

### Statistical analysis

In all experiments, data were evaluated for statistical significance using one-way ANOVA analysis of variance followed by Q-test. A difference was considered significant if the *P *value was less than 0.05.

## Results

### Effects of casein injection on the levels of pro-inflammatory cytokines

**Figure 1:**
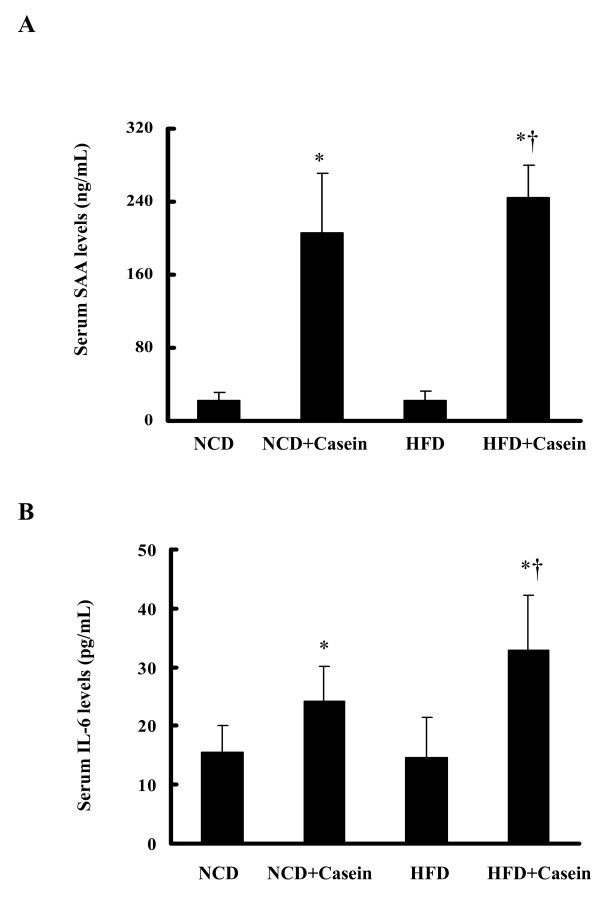
**Levels of inflammatory cytokines in the serum of C57BL/6J mice **. Mice fed with normal chow diet (NCD), NCD plus casein injection (NCD+Casein), high fat diet (HFD) and HFD plus casein injection (HFD+Casein) were culled 18 weeks after first injection. The levels of SAA (A) and IL-6 (B) in the serum of C57BL/6J mice were examined by enzyme-linked immunosorbent assay. Results represent the mean ± SD (n = 6). * P < 0.05 versus NCD, † P < 0.05 versus HFD.

We induced a chronic systemic inflammation by subcutaneous injection of 10% casein in C57BL/6J mice. There were significant increases of SAA and IL-6 in casein-injected mice fed either NCD or HFD compared with the respective controls (Figure 1A and 1B), suggesting that chronic systemic inflammation was successfully induced in C57BL/6J mice.

### Effects of chronic systemic inflammation on ectopic lipid deposition of C57BL/6J mice

**Figure 2:**
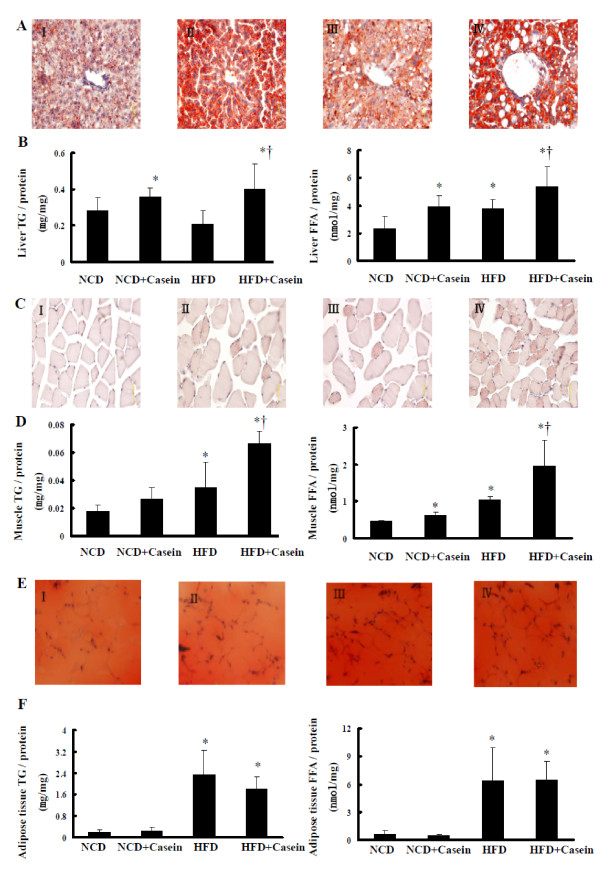
**Effects of chronic systemic inflammation on lipid accumulation in liver, muscle and white adipose tissue from C57BL/6J mice **. Mice fed with normal chow diet (NCD), NCD plus casein injection (NCD+Casein), high fat diet (HFD) and HFD plus casein injection (HFD+Casein) were culled 18 weeks after first injection. The lipid accumulation in tissues was checked by Oil Red O staining (A, C, E, original magnification × 400) using 5-μm-thick sections and quantitative lipid measurements of tissues (B, D, F). The concentrations of TG and FFA in tissues of C57BL/6J mice were measured as described in Materials and Methods and results represent the mean ± SD (n = 6), * P < 0.05 versus NCD, † P < 0.05 versus HFD. (A (I-IV)) Oil Red O staining of liver. (B) The concentrations of TG (left) and FFA (right) in liver. (C (I-IV)) Oil Red O staining of muscle. (D) The concentrations of TG (left) and FFA (right) in muscle. (E (I-IV)) Oil Red O staining of white adipose tissue. (F) The concentrations of TG (left) and FFA (right) in white adipose tissue.

Oil Red O staining showed that a HFD for 18 weeks induced lipid droplet accumulation in the liver (Figure 2A III *vs. *2A I), muscle (Figure 2B III *vs. *2B I), and white adipose tissues (Figure 2C III *vs. *2C I) compared with NCD group of C57BL/6J mice. Chronic systemic inflammation induced by casein injection exacerbated lipid accumulation in the liver from mice fed with NCD (Figure 2A II *vs. *2A I) and HFD (Figure 2A IV *vs. *2A III) as evidenced by Oil Red O staining and increased the index of liver weight/body weight (Table 2). Chronic systemic inflammation also increased lipid accumulation in the muscle from mice fed with NCD and HFD, with the appearance of massive lipid vacuoles compare to the respective controls (Figure 2C II *vs. *2C I and 2C IV *vs. *2C III). Interestingly, lipid droplet accumulation has no obvious change in while adipose tissue in the casein-injected mice fed with NCD or HFD (Figure 2E, I-IV). Quantitative assay further demonstrated that casein injection increased TG and FFA levels in muscle, to a greater degree in liver, and has no effect on TG and FFA levels in white adipose tissue of C57BL/6J mice (Figure 2B, D and 2F).

**Table 2:** Index of liver/body weights of C57BL/6J mice

Gram (n = 6)	NCD	NCD + Casein	HFD	HFD + Casein
Liver/body weights	0.04 ± 0.008	0.04 ± 0.002	0.05 ± 0.01*	0.06 ± 0.01*

* P < 0.05 versus NCD

### Effects of inflammation stress on lipogenesis in all tissues and lipolysis gene expression in white adipose tissue of C57BL/6J mice

To investigate potential mechanisms of the ectopic lipid deposition, we evaluated the effects of chronic systemic inflammation on the gene and protein expression of SREBP1, FAS, ACCα in liver, muscle and white adipose tissue of the C57BL/6J mice.

**Figure 3:**
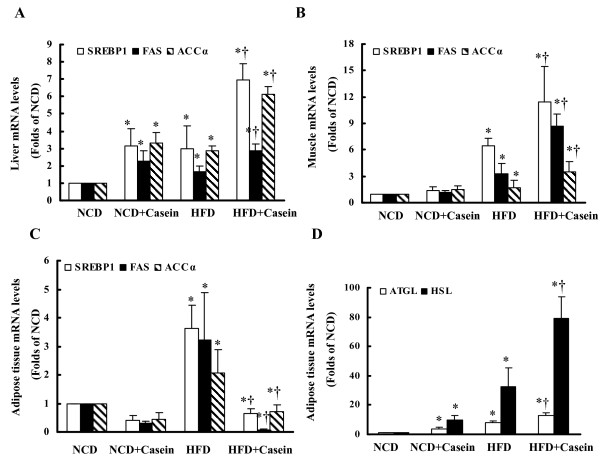
**Effects of chronic systemic inflammation on the mRNA expression of lipogenesis and lipolysis in liver, muscle and white adipose tissue of C57BL/6J mice **. Mice fed with normal chow diet (NCD), NCD plus casein injection (NCD+Casein), high fat diet (HFD) and HFD plus casein injection (HFD+Casein) were culled 18 weeks after first injection. The mRNA expression was determined by real-time PCR, 18s rRNA served as the housekeeping gene. (A, B, C) The mRNA expression of SREBP1, FAS and ACCα in liver, muscle and white adipose tissue of C57BL/6J mice. (D) The mRNA expression of ATGL and HSL in white adipose tissue of C57BL/6J mice. Results represent the mean ± SD (n = 6). * P < 0.05 versus NCD, † P < 0.05 versus HFD.

**Figure 4:**
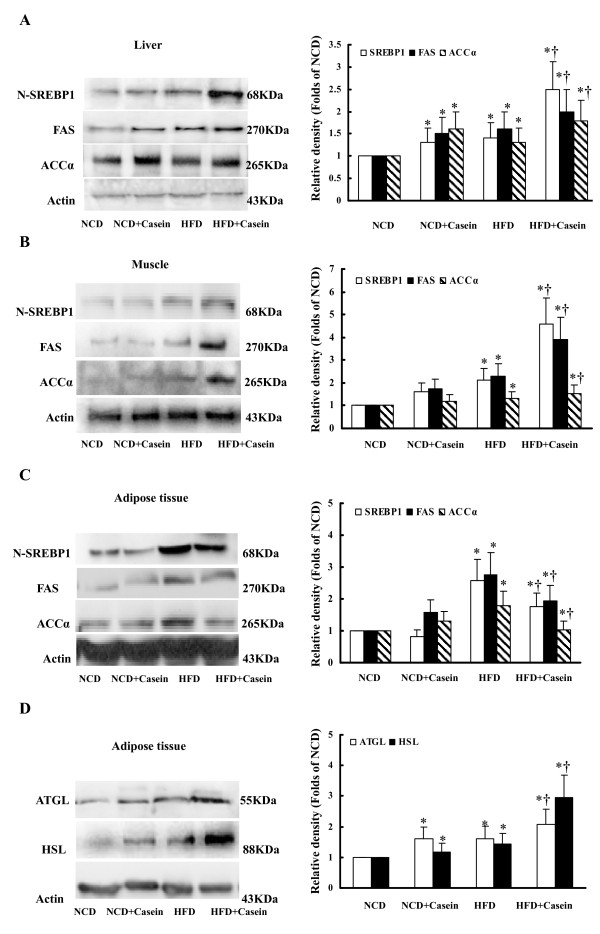
**Effects of chronic systemic inflammation on the protein expression of lipogenesis and lipolysis in liver, muscle and white adipose tissue of C57BL/6J mice **. Mice fed with normal chow diet (NCD), NCD plus casein injection (NCD+Casein), high fat diet (HFD) and HFD plus casein injection (HFD+Casein) were culled 18 weeks after first injection. The protein levels were determined by western blotting and β-actin served as the internal reference. (A, B, C) The protein levels of SREBP1, FAS and ACCα in liver, muscle and white adipose tissue of C57BL/6J mice. (D) The protein expression of ATGL and HSL in white adipose tissue of C57BL/6J mice. The histogram represents mean ± SD of the densitometric scans for protein bands from four experiments, normalized by comparison with β-actin, and expressed as a percentage of control. * P < 0.05 versus NCD, † P < 0.05 versus HFD.

Casein injection increased the mRNA (Figure 3A, B) and protein (Figure 4A, B) expression of SREBP1, FAS and ACCα in liver and muscle, especially in the mice fed with HFD, while it decreased mRNA and protein expression of these lipogenic molecules in white adipose tissue (Figure 3C and 4C). Interestingly, chronic systemic inflammation increased mRNA (Figure 3D) and protein (Figure 4D) expression of ATGL and HSL in white adipose tissue.

### Effects of inflammation stress on the levels of glucose, insulin and lipid in the serum of C57BL/6J mice

**Figure 5:**
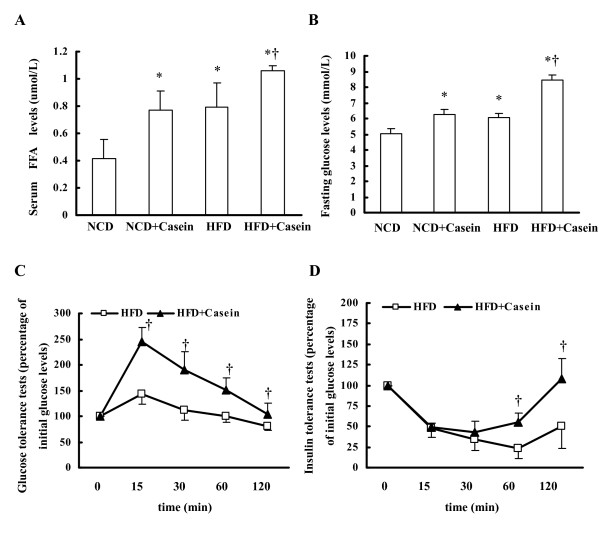
**Effects of chronic systemic inflammation on serum FFA concentrations, glucose and insulin resistance of C57BL/6J mice **. (A) FFA concentrations in the serum of C57BL/6J mice. (B) The fasting blood glucose concentrations in the C57BL/6J mice were measured as described in Materials and Methods. * P < 0.05 versus NCD, † P < 0.05 versus HFD. (C) Glucose tolerance tests (glucose-stimulated blood glucose concentrations) performed after 18 weeks of treatment in HFD group (white square, n = 6) and HFD plus casein injection mice (black triangle, n = 6). Results of each group were normalized by their initial blood glucose (before glucose stimulation). (D) Insulin tolerance tests performed at the end of experiments in C57BL/6J mice in HFD group (white square, n = 6) and HFD plus casein injection mice (black triangle, n = 6). Results of each group were normalized by their initial blood glucose (before insulin stimulation). Results represent the mean ± SD (n = 6). † P < 0.05 versus HFD.

Serum lipid assay demonstrated that levels of FFA in the casein-injected mice fed with NCD or HFD were increased compared with the respective controls (Figure 5A), suggesting that chronic systemic inflammation results in dyslipidemia in C57BL/6J mice. The fasting glucose levels in HFD plus casein injection group were increased compared to HFD alone group (Figure 5B). After glucose loading, blood glucose concentrations in HFD plus casein injection group were persistently higher than HFD group (Figure 5C), suggesting that chronic systemic inflammation induced glucose intolerance in C57BL/6J mice. After insulin challenge, blood glucose levels initially were decreased, but increased faster and higher in HFD plus casein injection group than in HFD group after 30 min, suggesting that chronic systemic inflammation decreased insulin sensitivity in C57BL/6J mice (Figure 5D).

## Discussion

Chronic systemic inflammation plays an important role in pathogenesis of multiple metabolic disorders, including insulin resistance, T2DM, NAFLD, atherosclerosis, obesity and dyslipidemia [15,16]. In this study, we use subcutaneous injection of casein to induce chronic systemic inflammation in C57BL/6J mice fed with NCD or HFD to investigate if inflammation causes ectopic lipid deposition in nonadipose tissues. The casein injection model has been used in studies of atherosclerosis and liver steatosis [17]. Compared to other sole cytokine-treated models, casein-injection induces multiple cytokines release which is more likely to mimic chronic systemic inflammatory states observed in patients with inflammatory diseases [18]. It is well-accepted that both SAA and IL-6 are good inflammatory markers for systemic and hepatic inflammatory stress. Our results showed that casein injection significantly increased serum levels of SAA and IL-6, suggesting that chronic systemic inflammation was successfully induced in C57BL/6J mice.

Normally, FFA delivered to adipose tissue is converted to TG for storage during states of nutrient abundance. Therefore white adipose tissue has a unique capacity to store large amounts of excess lipid, while nonadipose tissues in which FFA was utilized have a limited capacity for storage of lipids [19,20]. Adipose lipids also can be released into circulation to form albumin/FFA complexes, which allow FFA transport into nonadipose tissues, such as muscle, heart, kidney and liver for β oxidation on demand. Insulin regulates this process to maintain FFA homeostasis in balance. As liver and muscle are main insulin-sensitive and ectopic lipid accumulated tissues [21], we examined the effect of chronic systemic inflammation on lipid accumulation in these tissues. Oil Red O staining and quantitative assays demonstrated massive TG and FFA accumulation in the liver and muscle in casein-injected mice fed with NCD or HFD, suggesting a strong association between ectopic lipid deposition and chronic systemic inflammation. We also observed that the increases of TG and FFA contents induced by inflammation are more obvious in liver than in muscle in both NCD and HFD fed mice, which may attribute to the preferential ability of liver in a tissue-specific manner for lipogenesis. Interestingly, in the same experimental setting, chronic systemic inflammation has no obvious effects on the lipid contents in white adipose tissues.

Furthermore, we studied the expression of key molecules involved in lipogenesis to explore potential mechanisms of accelerated lipid accumulation induced by casein injection. Our results showed that chronic systemic inflammation up-regulated mRNA and protein expression of SREBP1, ACCα and FAS in liver and muscle, while decreasing the expression of these genes in adipose tissue. These data suggest that inflammation tissue-specifically switches on lipogenesis in nonadipose tissues, especially in liver while switches off lipogenesis in adipose tissue.

Lipid metabolism in adipose tissue is governed by lipogenesis and lipolysis. The lipolysis of white adipose tissue is complicated multi-step processes which performed from TG to glycerol and FFA. Previous studies discovered that enzyme ATGL seems to be responsible for the bulk of triacylglycerol hydrolase activity in various cells and ATGL has low affinity to diacylglycerides and monoacylglycerides. The major diacylglyceride lipase in adipocytes is HSL. Monoacylglyceride products of HSL are hydrolyzed by constitutively active monoacylglyceride lipase. We showed that casein injection increased both mRNA and protein expression of ATGL and HSL in white adipose tissue, suggesting that chronic systemic inflammation disrupts lipid metabolism by increasing lipolysis in addition to an impaired lipogenesis in adipose tissue.

Ectopic lipid deposition is closely related to the development of insulin resistance and dyslipidaemia [22]. We evaluated serum lipids, insulin and glucose tolerance in the mice. Our data demonstrated that chronic systemic inflammation induced by casein injection exacerbated hyperlipidaemia in C57BL/6J mice. Accordingly, in this study, we also observed that casein injection induced hyperglycemia and insulin resistance in C57BL/6J mice. In this context, insulin cannot effectively suppress adipose tissue lipid hydrolysis which in turn increased adipose tissue lipolysis.

## Conclusions

This study demonstrated that chronic systemic inflammation increased lipogenesis in nonadipose tissues, while it down-regulated lipogenesis and up-regulated lipolysis in white adipose tissues, resulting in ectopic lipid deposition in nonadipose tissues. The deposition of large amounts of lipids in nonadipose tissue may trigger ER stress, oxidative stress and apoptosis, thereby causing tissue injuries. The cytokines or chemokines released by injured cells further exacerbate inflammation, lipid accumulation and insulin resistance in a vicious cycle [23,24]. The inflamed model established in the experiments may be useful for studying the organ cross-talks for FFA homeostasis and the mechanisms of ectopic lipid deposition induced by chronic systemic inflammation, especially rising from chronic inflammatory diseases characterized by an increased CRP level, as in Crohn's disease [25], autoimmune diseases [26], T2DM, and progressive NAFLD [27].

## Abbreviations

ACCα: Acetyl-CoA carboxylase alpha; ATGL: Adipose triglyceride lipase; CRP: C-reactive protein; DG: Diglycerides; ER: Endoplasmic reticulum; FAS: Fatty acid synthase; FFA: Free fatty acid; HFD: High fat diet; HSL: Hormone-sensitive lipase; IL-6: Interleukin-6; NAFLD: Non-alcoholic fatty liver disease; NCD: Normal chow diet; PCR: Polymerase chain reaction; SAA: Serum amyloid A protein; SREBPs: Sterol regulatory binding proteins; SREBP1: Sterol regulatory binding protein 1; SREBP2: Sterol regulatory binding protein 2; T2DM: Type 2 diabetes; TNF-α: Tumor necrosis factor alpha; TG: Triglyceride.

## Competing interests

The authors declare that they have no competing interests.

## Grants

This study was supported by the National Natural Science Foundation of China (30871159, 30971389, 81070631, 81070317 and Key Program, No. 81030008), the Ministry of Education of China (20105503120004), Chongqing Education Committee (KJ110302), the Moorhead Trust, the Royal Free Hospital Special Trustees Grant-115.

## Authors' contributions

MM and LZ carried out experiments, data collection, performed the statistical analysis and drafted the manuscript. QL participated in the coordination of the animal experiments. AIH , ZV , JFM, SHZ, SHP and QFL helped to draft the manuscript. YXC and XZR participated in the design of the experiments and helped to draft the manuscript. All authors read and approved the final manuscript.
